# Robust, site-specifically immobilized phenylalanine ammonia-lyases for the enantioselective ammonia addition of cinnamic acids†

**DOI:** 10.1039/d1cy00195g

**Published:** 2021-06-29

**Authors:** Krisztina Boros, Mădălina Elena Moisă, Csaba Levente Nagy, Csaba Paizs, Monica Ioana Toşa, László Csaba Bencze

**Affiliations:** Enzymology and Applied Biocatalysis Research Center, Faculty of Chemistry and Chemical Engineering, Babeş-Bolyai University Arany János Str. 11 RO-400028 Cluj-Napoca Romania cslbencze@chem.ubbcluj.ro

## Abstract

Phenylalanine ammonia-lyases (PALs) catalyse the non-oxidative deamination of l-phenylalanine to *trans*-cinnamic acid, while in the presence of high ammonia concentration, the synthetically attractive reverse reaction occurs. Although they have been intensively studied, the wider application of PALs for the large scale synthesis of non-natural amino acids is still rather limited, mainly due to the decreased operational stability of PALs under the high ammonia concentration conditions of ammonia addition. Herein, we describe the development of a highly stable and active immobilized PAL-biocatalyst obtained through site-specific covalent immobilization onto single-walled carbon nanotubes (SWCNTs), employing maleimide/thiol coupling of engineered enzymes containing surficial Cys residues. The immobilization method afforded robust biocatalysts (by strong covalent attachment to the support) and allowed modulation of enzymatic activity (by proper selection of binding site, controlling the orientation of the enzyme attached to the support). The novel biocatalysts were investigated in PAL-catalyzed reactions, focusing on the synthetically challenging ammonia addition reaction. The optimization of the immobilization (enzyme load) and reaction conditions (substrate : biocatalyst ratio, ammonia source, reaction temperature) involving the best performing biocatalyst SWCNT_NH_2__-SS-*Pc*PAL was performed. The biocatalyst, under the optimal reaction conditions, showed high catalytic efficiency, providing excellent conversion (*c* ∼90% in 10 h) of cinnamic acid into l-Phe, and more importantly, possesses high operational stability, maintaining its high efficiency over >7 reaction cycles. Moreover, the site-specifically immobilized *Pc*PAL L134A/S614C and *Pc*PAL I460V/S614C variants were successfully applied in the synthesis of several l-phenylalanine analogues of high synthetic value, providing perspectives for the efficient replacement of classical synthetic methods for l-phenylalanines with a mild, selective and eco-friendly enzymatic alternative.

## Abstract 2

Phenylalanine ammonia-lyases (PALs), site-specifically immobilized on single-walled carbon nanotubes (SWCNT), provide robust biocatalysts for the synthetically important ammonia additions onto cinnamic acids, yielding l-phenylalanines.

## Graphical abstract

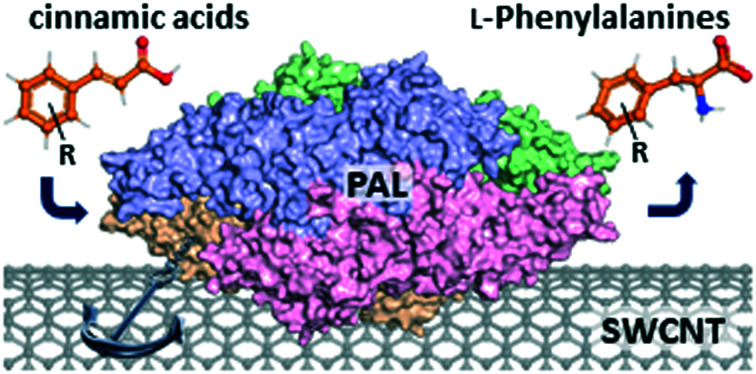

## Introduction

Recently, there has been tremendous progress in the development of biotransformation-based synthetic procedures. An increasing number of chemical reactions have their enzymatic analogues provided with high (chemo-, regio- and enantio-) selectivity and efficiency, removing the need for toxic reagents.^1,2^ Accordingly, enzymes serve as biocompatible, biodegradable, environmentally friendly and economical alternatives to classical catalysts^3^ and significantly contribute to the development of green and sustainable chemical/biotechnological manufacturing processes.

**Fig. 1:**
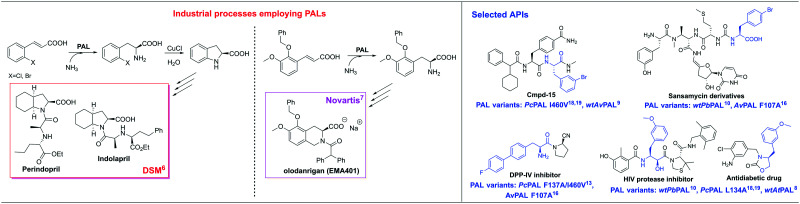
Examples of PAL-based industrial processes and APIs containing l-phenylalanine derivatives.

Aromatic amino acid ammonia-lyases, especially phenylalanine ammonia-lyases (PALs EC 4.3.1.24; PAL/TALs with dual phenylalanine and tyrosine ammonia-lyase activities, EC 4.3.1.25), are among the most attractive biocatalysts for the production of optically pure phenylalanine analogues, important building blocks in pharmaceutical and synthetic chemistry (Fig. 1).^4^ The PAL-based ammonia addition reaction of cinnamic acid derivatives has multiple advantages over other synthetic methods, such as a 100% atom economy, high enantioselectivity, low-cost, easily accessible substrates, and no requirement for a cofactor or its regeneration.^4,5^ PALs are already used in established industrial processes, such as the multi-ton scale production of (*S*)-2,3-dihydro-1*H*-indole-2-carboxylic acid by DSM^6^ or of (3*S*)-5-(benzyloxy)-2-(diphenylacetyl)-6-methoxy-1,2,3,4-tetrahydroisoquinoline-3-carboxylate (EMA401) by Novartis^7^ (Fig. 1). Consequently, within the last decade, PALs have been intensively studied^4,8–11^ and the current wave of protein engineering resulted in variants with extended substrate scopes^12–14^ and increased catalytic activity.^15–20^ Aside from enhanced activity and high selectivity, stability is a crucial requirement that affects the efficiency of a biocatalyst, which, in the case of the PAL-catalysed ammonia addition reactions, is challenging to accomplish, due to the required high 4–6 M NH_3_ content of the reaction mixture.

Through enzyme immobilization, the operational stability of biocatalysts can be significantly improved, as well as their facile separation from the reaction mixture, which enables their recovery and reuse in further reaction cycles.^3,21^ Accordingly, immobilized enzymes represent an increasing percentage of the industrially employed biocatalysts. Several attempts focused on the immobilization of PALs in order to increase their operational/thermal stability. Although techniques like microencapsulation^22^ and cross-linking with glycerol diglycidyl ether^23^ led to biocatalysts with higher thermal and storage stability, none of these preparates proved to be stable in the ammonia addition reaction, hindering their recycling, while their inappropriate shape, particle size, and mechanical properties disabled their use in continuous flow packed-bed reactors.^24^ Among all types of immobilization, the most robust approach is the covalent attachment of a protein to a support.^3,25^ A recombinant PAL from *Petroselinum crispum* (*Pc*PAL) covalently immobilized on carboxy- and amino-functionalized single-walled carbon nanotubes (SWCNTs) proved to be highly stable and durable biocatalysts in the case of the ammonia elimination reaction.^24,26^ Nevertheless, during the synthetically more important reverse ammonia addition reaction, the biocatalysts presented high stability/recyclability only upon rigorous catalyst reconditioning, consisting of a 2 hour washing procedure.^26^ The covalent attachment of *Pc*PAL to magnetic nanoparticles (MNPs) was also accomplished;^27^ however, the enzyme preparation showed low activity and stability in the ammonia addition reaction. The combination of covalent binding and chelating binding of *Pc*PAL onto magnetic nanoparticles significantly improved its recyclability, with the biocatalyst maintaining 90% of its initial activity over 5 cycles,^28^ but it was notable that in the case of SWCNT-PALs,^24,26^ a significant activity drop in ammonia addition occurred only after >5 reaction cycles.

The main drawbacks of covalent immobilization are the unwanted bonding orientations and the unspecific multipoint attachments, which can lead to the rigidization of the biomolecule, resulting in a significant activity loss compared to the free enzyme.^3,25^ In order to overcome these difficulties, various site-specific approaches have been developed for the covalent fixation of proteins,^25,29–33^ enabling the attachment of proteins to an appropriate support from a single, priorly selected point of their structure. The linkage can be achieved also through enzyme-mediated binding, such as in the case of using the sortase A enzyme as a fusion partner^34^ or using different tags such as SNAP-tag,^35^ HaloTag^36^ or CLIP-tag.^37^ The site-specific incorporation of a non-natural amino acid, with specific reactivity, is an attractive approach^29,30^ to control the site within the protein structure for the covalent linkage to the support; however, the required bioorthogonal expression system decreases the accessibility of this procedure.^38^ A more accessible site-oriented technique relies on the maleimide-mediated alkylation of the thiol group of cysteine (Cys) introduced in a well-defined position on the surface of the enzyme, presuming the target enzyme does not contain other surficial cysteine residues.^31–33^

In this study, following our aim to create robust PAL biocatalysts for the synthetically valuable ammonia addition reactions, we focused on the development of site-specifically immobilized PALs employing the maleimide/thiol coupling approach. Through fine-tuning of the immobilization procedure and reaction conditions, a PAL-biocatalyst with an unprecedentedly high operational stability and recyclability has been developed, leading to highly efficient ammonia additions of cinnamic acids.

## Results and discussion

**Fig. 2:**
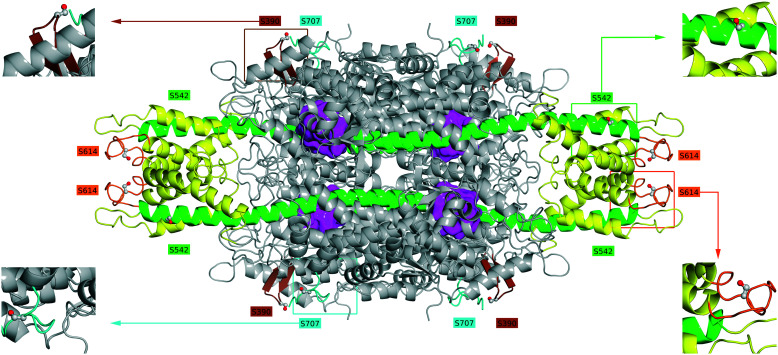
The surficial serine residues from the tetrameric structure of *Pc*PAL (PDB ID: 6F6T^40^) selected for their replacement with cysteine. All selected residues are located distal to the four catalytic sites (magenta) of the homotetramer. The loop containing S614 (orange) and the helix containing S542 (green) are part of the insertion domain (marked in green) involving residues 527–648. Residue 390 is located within a hairpin connecting two antiparallel β-sheets (brown), while residue 707 is part of the C-terminal end loop of *Pc*PAL (blue).

Using the crystal structure of *Pc*PAL (PDB ID: 1W27,^39^6RGS,^18^6F6T^40^), several surficial serine residues (Ser) positioned far from the catalytic site (Fig. 2) were selected for their individual replacement to cysteine, resulting in four *Pc*PAL variants with single mutations S390C, S542C, S614C, and S707C. The two original surficial cysteines, C704 and C716, of *Pc*PAL have been already replaced with Ser in our previous studies,^18,41^ thus ensuring that the site-specific immobilization, employing the maleimide–thiol coupling, proceeds only in one of the four positions of the newly introduced surficial Cys residues. The purified forms of all four *Pc*PAL variants presented similar catalytic activity (Table S2†) and thermal denaturing profiles (Fig. S1†) those of the wild-type enzyme, showing that the mutations did not affect the activity and the overall protein fold.

**Fig. 3:**
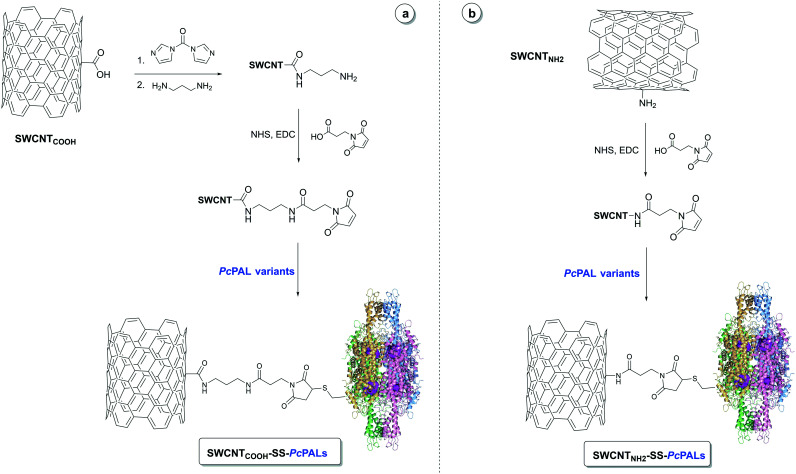
Site-controlled covalent immobilization of *Pc*PAL on the a) SWCNT_COOH_ and b) SWCNT_NH_2__ supports.

In our previous studies, several supports, such as differently functionalized silane-coated magnetic nanoparticles,^27,42^ single-walled carbon nanotubes,^24,26^ functionalized graphene, chitosan and commercial epoxy beads from Chiralvision,^[unpublished results]^ have been tested for the covalent immobilization of PALs. Generally, all immobilized preparations underperformed under the harsh conditions of ammonia additions in comparison with those under ammonia eliminations, with the most promising activity and operational stability being achieved with the use of SWCNTs functionalized with amino groups as a support.^26^ Despite the increased attention for the industrial applicability of PAL-based procedures, no other studies reported immobilized PAL preparations with high operational stability in ammonia addition, while an attempt described within the PAL process development for EMA401 (ref. 7) has supported the existence of such interests. Accordingly, using SWCNT-based supports, the best performing in our previous studies, a site-oriented immobilization procedure was established using a maleimide moiety containing linker that was attached directly to amino-functionalized carbon nanotubes (SWCNT_NH_2__) or to carboxy-functionalized carbon nanotubes (SWCNT_COOH_), priorly activated with carbonyldiimidazole and functionalized with 1,3-diaminopropane (Fig. 3). It is worth mentioning that both supports are commercially available, also in bulk quantities, and thus, instead of the tedious microwave assisted NH_2_-functionalization of SWCNTs, as performed in our previous study,^26^ we could use more accessible commercial supports for both carboxy- and amino-functionalized SWCNTs. The immobilization yield was high in all cases (Table S4†), with >95% of the added *Pc*PALs being bound to the supports.

**Fig. 4:**
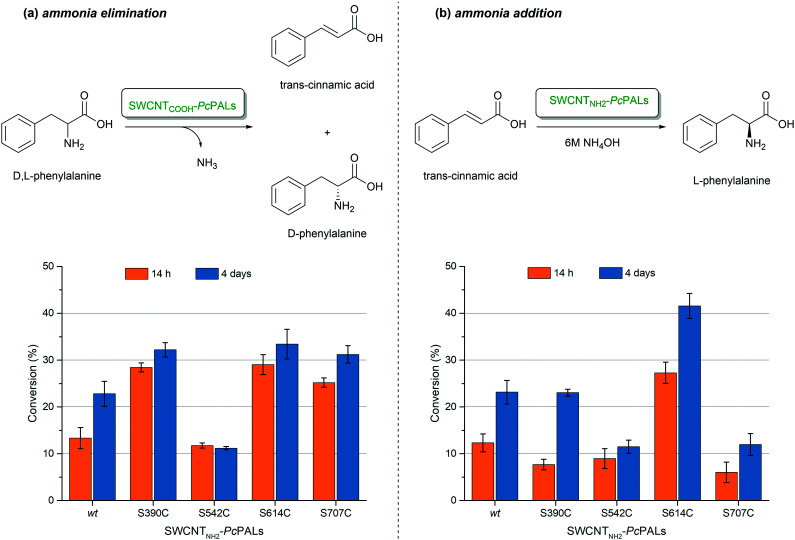
Conversions of a) the ammonia elimination reaction starting from the racemic d,l-Phe and of b) the ammonia additions from *trans*-cinnamic acid using, as biocatalysts, the four site-specifically immobilized *Pc*PAL variants S390C, S542C, S614C, and S707C and the non-specifically, covalently immobilized *wt-Pc*PAL.

Further, the immobilized SWCNT-PALs were tested in both ammonia elimination and addition reactions (Fig. 4). While the PALs site-specifically immobilized on carboxy-functionalized carbon nanotubes (SWCNT_COOH_-SS-PALs) provided higher conversions in the elimination route, SWCNT_NH_2__-SS-PALs, obtained through immobilization on amino-functionalized carbon nanotubes, showed higher efficiency in the ammonia addition reaction (Table S6†). This is in accordance with our previous results, where PAL covalently immobilized on amino-functionalized carbon nanotubes SWCNT_NH_2__-GDE-*wtPc*PAL showed higher activity in ammonia additions than its SWCNT_COOH_-GDE-*wtPc*PAL counterpart, immobilized on carboxy-functionalized nanotubes.^26^ Next, we compared the catalytic performance of the novel site-specifically immobilized PALs with that of their priorly developed covalently, but non-specifically, immobilized counterparts, SWCNT_COOH_-GDE-*wtPc*PAL^24^ and SWCNT_NH_2__-GDE-*wtPc*PAL.^26^

In the case of the ammonia elimination reaction, applicable for the kinetic resolution of racemic phenylalanine, three of the four site-specifically immobilized biocatalysts provided higher conversions, and thus have increased catalytic efficiency compared to the non-specifically immobilized *Pc*PAL (Fig. 4a).

In the ammonia addition reactions, performed at high ammonia concentration (6 M NH_4_OH), the site-specifically immobilized *Pc*PAL S614C showed a significantly higher enzymatic activity than the rest of the enzyme preparations obtained through site-specific immobilization at other positions (Ser390, Ser542, Ser707) or the priorly developed non-specifically, covalently immobilized biocatalyst SWCNT_NH_2__-GDE-*wtPc*PAL (Fig. 4b). All PAL-based biocatalysts showed complete enantioselectivity towards the l-enantiomer in both reaction routes (Fig. S5†).

Interestingly, residue 614 is located at a loop connecting two helices at the top of the additional insertion domain of eukaryotic PALs, which extends above and below the main body of the structure and is composed of residues 527–648 in the case of *Pc*PAL^43^ (Fig. 2). The high flexibility of this additional region is considered to provide the lower thermostability/protease stability of the eukaryotic PALs in comparison with prokaryotic PALs of a shorter sequence and more compact structure.^43–45^

Within the crystal structure of the *Pc*PAL I460V variant (PDB ID: 6RGS^18^) complexed with *p*-MeO-cinnamic acid, a substrate analogue of the ammonia addition reaction, the loop containing S614 appears to have insufficient electron density, supporting its high flexibility, which might be reduced through its covalent binding to the support, thus providing increased operational stability to the SWCNT_NH_2__-SS-PAL/S614C biocatalyst. In *Pc*PAL structures complexed with inhibitor-type ligands such as DTT (PDB ID: 1W27 (ref. 39)) and S-APPA (PDB ID: 6F6T^40^) the loop containing residue 614 is visible (Fig. S6†). Besides, the high ammonia content in ammonia addition provides different conditions from those employed in protein crystallizations and alters the thermal denaturing profile of *Pc*PAL,^13^ decreasing its *T*_m_ by 10–12 °C; thus, presumably, the conformation of the flexible insertion domain is highly affected. Residue S542 is also part of the insertion domain, but is located in a high stability region, being part of the longest helix (residues 496–547) entering the core structure, which also covers the catalytic site. Residues 390 and 707 are not part of the insertion domain and are located within a hairpin connecting two antiparallel β-sheets, or within the loop of the C-terminal end of PALs, associated with their aggregation tendency.^45^

Further, considering our aim to develop an immobilized PAL-biocatalyst for the synthetically valuable ammonia addition reaction and the catalytic superiority of the biocatalyst based on the *Pc*PAL S614C variant, we focused on the optimization of the immobilization procedure and reaction conditions to improve the catalytic performance of the SWCNT_NH_2__-SS-*Pc*PAL/S614C enzyme preparate (further referred to as SWCNT_NH_2__-SS-*Pc*PAL).

### The influence of the biocatalyst : substrate ratio and enzyme loading on the conversion

**Fig. 5:**
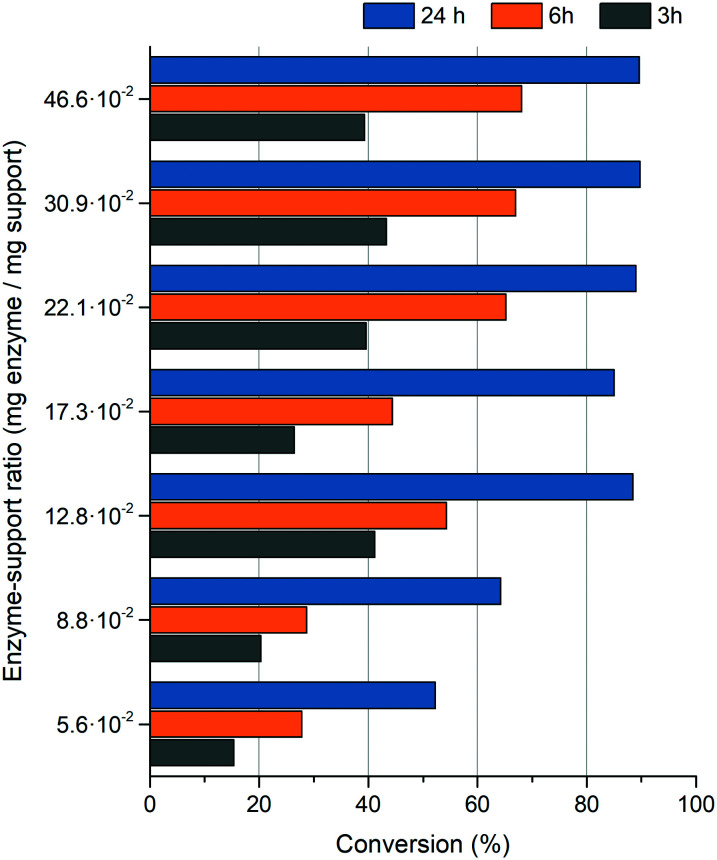
Conversions of the ammonia addition reactions of *trans*-cinnamic acid after 3, 6 and 24 hours of reaction time, using the SWCNT_NH_2__-SS-*Pc*PAL enzyme preparate with different enzyme loads.

Initially, in the first optimization step, the influence of the biocatalyst load on the reaction system was tested. By increasing the amount of the SWCNT_NH_2__-SS-*Pc*PAL biocatalyst up to five-fold in the reaction, the conversion values showed a corresponding growing tendency (Fig. S7†). Therefore, we tested different enzyme loads of the immobilization preparate with the aim of maximizing the enzyme : support ratio, which would also provide higher conversions. Enzyme loads higher than ∼0.5 (mg enzyme per mg support) resulted in immobilization yields lower than ∼80% and were omitted from further experiments in order to avoid the loss of the non-binding fraction of the purified enzyme. The obtained conversion values suggest that the optimal enzyme load is ∼0.13. Immobilized preparations with higher enzyme content did not provide a significant increase of the conversions that would outweigh the use of an increased amount of the high value, purified enzyme (Fig. 5).

### The effect of the ammonia source

The effect of the reaction medium, serving both as an ammonia source and reaction buffer, was tested using the SWCNT_NH_2__-SS-*Pc*PAL biocatalyst with the priorly determined optimal enzyme/support ratio. In previous PAL-mediated biotransformations, since 2 M and 4 M ammonium carbamate or 6 M NH_4_OH solutions proved to be an optimal ammonia source,^9,13,19^ we tested different concentrations of the two ammonia sources as a reaction medium, considering that ammonium carbamate can provide two molecules of ammonia, instead of one provided by ammonium hydroxide (Table 1).

**Table 1:** Conversions for the ammonia addition of cinnamic acid catalyzed by SWCNT_NH_2__-SS-*Pc*PAL

	Ammonia source						
	NH_2_CO_2_NH_4_				NH_4_OH		
Conc. (M)	1	2	3	4	2	4	6
Conv. (%)	74.7	87.8	92.6	94.1	61.7	80.2	84.9

As expected, the increased ammonia concentration resulted in higher conversions, shifting the ammonia addition–ammonia elimination equilibrium towards the amino acid production, while the use of ammonium carbamate as the ammonia source was also more advantageous than the use of NH_4_OH solutions, probably due to its lower ionic strength that might contribute to an increased biocatalyst stability. Accordingly, previous reports also described ammonium carbamate as the optimal choice for an ammonia source for whole-cell mediated PAL reactions.^9,19^

### Recycling stability of the biocatalyst

SWCNT_NH_2__-GDE-*wtPc*PAL,^26^ with the most promising operational stability reported for the ammonia additions, requires, after each reaction cycle, a 2 hour phosphate buffer incubation/washing–reconditioning step to maintain its long-term activity. For a cost-effective PAL-based ammonia addition procedure, since the facile recyclability of the immobilized biocatalyst is essential, we tested the catalytic performance of SWCNT_NH_2__-SS-*Pc*PAL within several reaction cycles.

Due to the fact that the ammonia source and its concentration significantly affect not only the conversion values but also the biocatalyst's operational stability, the catalyst recycling tests were performed in both ammonium carbamate (Fig. S8†) and ammonium hydroxide solutions (Fig. S9†).

**Fig. 6:**
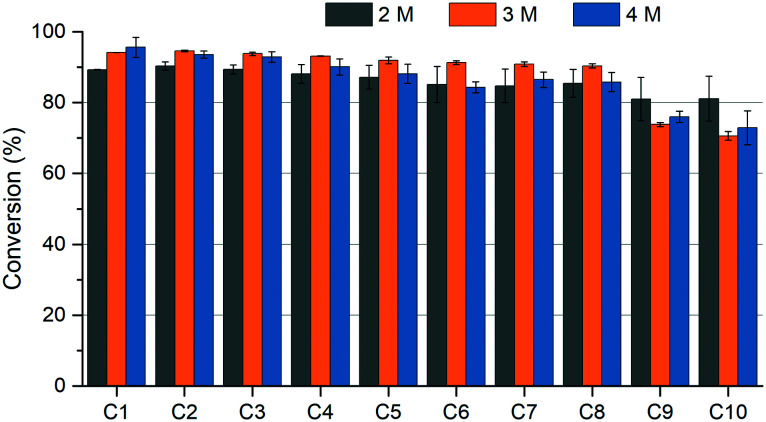
Recyclability of SWCNT_NH_2__-SS-*Pc*PAL in the ammonia addition reaction of cinnamic acid using ammonium carbamate (2–4 M; pH 9.6–10.0) as the ammonia source.

Using ammonium carbamate as a reaction medium, besides the higher conversions, also, better operational stability was obtained. After 8 reaction cycles, no significant decrease of the initial conversion was registered, with a ∼19% decrease being only observed starting from the 9th reaction cycle (Fig. S8†). Under the harsh reaction conditions of 6 M ammonium hydroxide, the site-specifically immobilized protein could be reused for 6 cycles without a significant loss of its initial activity (Fig. S9†), while the activity loss upon more intense catalyst recycling suggests that partial protein inactivation occurs. When testing the biocatalyst's recyclability in reaction media with different concentrations of ammonium carbamate, 2 M ammonium carbamate provided slightly higher operational stability for SWCNT_NH_2__-SS-*Pc*PAL than the corresponding 3 M and 4 M solutions, where the conversion dropped by 12–14% from the 9th reaction cycle (Fig. 6).

The results support that for the high operational stability of SWCNT_NH_2__-SS-*Pc*PAL, no reconditioning step is necessary. Notably, the recyclability of the reported SWCNT_NH_2__-GDE-PAL was tested under the harsher 6 M NH_4_OH reaction conditions, using 3-(thiophenyl-2-yl)acrylic acid as a substrate,^26^ hindering direct comparison with the current results. Therefore, for the unambiguous assessment of the superior operational stability of the site-specifically immobilized version, we also tested the recyclability of SWCNT_NH_2__-GDE-PAL under the optimal reaction conditions and enzyme load reported here. The activity differences observed in the biocatalyst screening step (Fig. 4) were maintained also in the case of using the higher, optimal enzyme load for both biocatalysts (Table S7†), supporting the higher activity of the site-specific PAL preparation. However, in ammonium carbamate, the operational stability of SWCNT_NH_2__-GDE-PAL was also improved in comparison with that reported in 6 M NH_4_OH.^26^ Besides the activity, the operational stability of SWCNT_NH_2__-SS-*Pc*PAL is still superior (Fig. S8†), most probably due to the combined effect of proper selection/control of the immobilization site and of the ammonium source.

### Effect of temperature

**Fig. 7:**
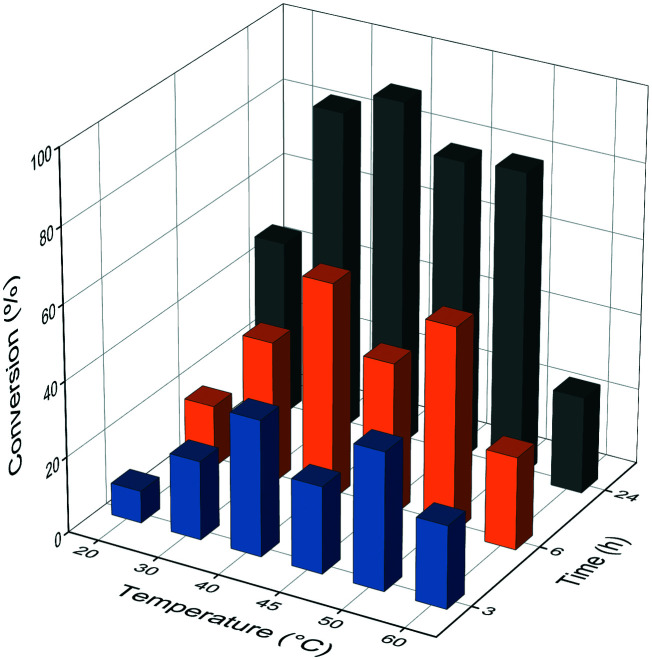
Effect of temperature on the conversion values of the ammonia addition reaction of *trans*-cinnamic acid, using the optimal reaction medium (3 M NH_2_CO_2_NH_4_) and biocatalyst load (0.12 mg enzyme per mg support). Samples were taken at different reaction times (3, 6 and 24 hours respectively).

Further, we tested the influence of the temperature on the ammonia addition reaction (Fig. 7). The optimal temperature in our case was 40 °C, reaching excellent ∼90% conversions after a 24 h reaction time. Interestingly, SWCNT_NH_2__-GDE-*Pc*PAL showed a different activity–temperature profile,^26^ with a local activity–conversion minimum at 50 °C, followed by an increasing tendency of conversion at higher temperatures. In our case, SWCNT_NH_2__-SS-*Pc*PAL provides low conversions at temperatures exceeding 50 °C. It was notable that in the case of SWCNT_NH_2__-GDE-*Pc*PAL, multipoint attachment was supposed,^26^ which might contribute to the maintenance of conformational stability during temperature increase, which can be ruled out in the case of the site-specific, single-point immobilization.

### Time–conversion profile

Finally, we determined the time–conversion profile of the SWCNT_NH_2__-SS-PAL catalyzed ammonia addition of *trans*-cinnamic acid performed under the optimal reaction conditions (Fig. S10†).

Excellent ∼90% conversion values were achieved already after 10 hours of reaction time, although a further increase of the conversion values with longer reaction times was not observed, suggesting that reaction equilibrium was established. As shown before, a further increase in conversion might be possible by increasing the ammonia content of the reaction buffer; however, this decreases the operational stability of the biocatalyst, showing that we approached the upper limits of the optimal conversion that can be ensured within several reaction cycles.

### Applicability for the synthesis of non-natural l-phenylalanines

**Fig. 8:**
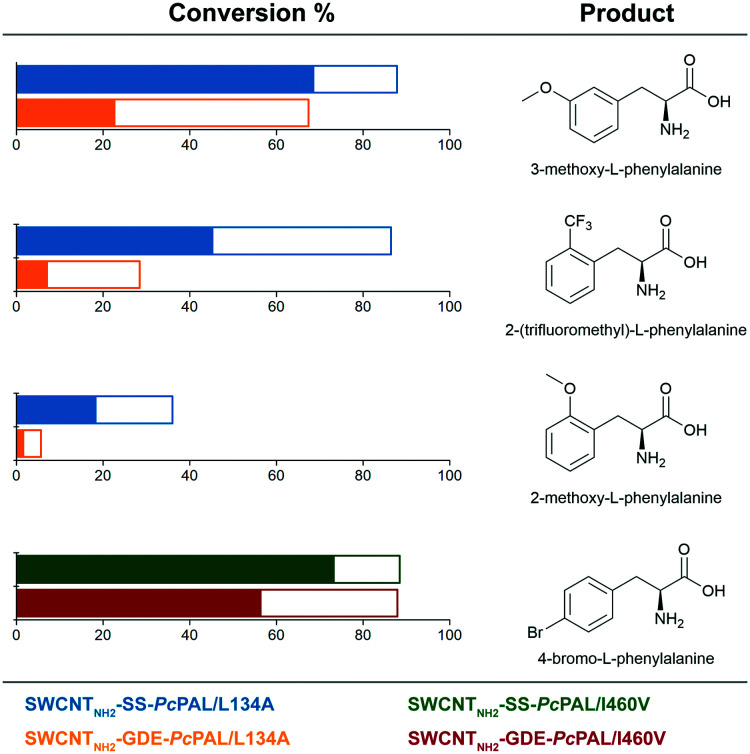
Ammonia addition reactions of cinnamic acid derivatives catalyzed by site-specifically and non-specifically immobilized engineered *Pc*PAL variants L134A and I460V. The color filled and empty bars represent the conversion values after 4 h and 20 h reaction times, respectively.

The immobilization procedure was applied for the engineered *Pc*PAL variants, *Pc*PAL L134A and *Pc*PAL I460V, of improved activity for non-natural substrates,^13,18^ thus resulting in l-phenylalanine analogues of economic importance *via* the ammonia addition reaction (Fig. 8).

The conversion values obtained with the site-specifically immobilized tailored PALs were compared with the conversions using their covalently, but non-specifically, immobilized counterparts. In accordance with the results presented earlier, all SWCNT_NH_2__-SS-PAL variants showed superior catalytic performance to that of the corresponding SWCNT_NH_2__-GDE-PALs (Fig. 8).

## Experimental

### Materials and methods

Both amino- and carboxy-functionalized single-walled carbon nanotubes (SWCNTs; ID = 0.8–1.6 nm, OD 1–2 nm, length = 5–30 μm) were purchased from Chengdu Organic Chemicals Co. Ltd (China). 1-Ethyl-3-(3-dimethylaminopropyl)carbodiimide hydrochloride (EDAC HCl) was a product of Molekula (Germany). *N*,*N′*-Carbonyldiimidazole (CDI), propane-1,3-diamine, *N*-hydroxysuccinimide (NHS), *N*-maleoyl-β-alanine and ammonium carbamate were products of Alfa Aesar (USA). Glycerol diglycidyl ether (GDE) and tris(hydroxymethyl)aminomethane (TRIS) were purchased from Sigma-Aldrich (USA). The Bradford reagent for determination of protein concentrations and ammonia solution (32%) were purchased from VWR (USA). Technical grade solvents such as methanol, dimethylformamide and dichloromethane were dried and/or freshly distilled prior to use, while HPLC-grade solvents were purchased from Promochem LGC Standards (Germany).

Site-directed mutagenesis for obtaining the mutant *Pc*PAL genes was performed using the corresponding mutagenic primers (Table S1†) and Phusion High Fidelity Polymerase (2 U μL^−1^) from Thermo Fisher Scientific in a 96-well Eppendorf Mastercycler ProS equipment. Plasmid extraction was performed using a GenElute Plasmid Miniprep Kit, available from Sigma-Aldrich. The enzyme immobilizations were carried out in a Heidolph Multi Reax shaker, while lyophilization of immobilized preparates was carried out with an ALPHA 1-2 LDPlus lyophilizer. UV-vis measurements were performed on an Agilent 8453 UV-vis spectrophotometer and a Tecan Spark 10 M microplate reader, using Corning 96-well Clear Flat Bottom UV-Transparent microplates. Enzymatic reactions were carried out in a Heidolph Multi Reax shaker, a Heidolph Vibramax 110 platform shaker coupled with a heating module or an Eppendorf ThermoMixer C. HPLC analysis of enzymatic reactions for determination of conversions and enantiomeric excesses was conducted on Agilent 1200 and 1100 Series instruments as priorly described^13,18^ and detailed in the ESI† (section 4).

### Protein expression, isolation, purification, and activity tests

The plasmids containing the *pcpal* genes with the desired mutation were transformed into *E. coli* Rosetta(DE3)pLysS competent cells by heat shock. The expression and purification of recombinant *Pc*PAL variants were performed according to our priorly optimized protocol.^41^ The purity of the isolated proteins was investigated through SDS-PAGE, while the enzymatic activity of the obtained *Pc*PAL variants was tested and compared with the activity of the wild-type *Pc*PAL by measuring the absorbance increase at 290 nm, corresponding to the *trans*-cinnamic acid specific absorbance maximum. A detailed description of the activity tests is included in ESI† section 2.

### Site-specific immobilization onto SWCNT_COOH_

20 mg of SWCNT_COOH_ was incubated with 3 mL of 200 mM CDI solution in CH_2_Cl_2_ overnight (or 4 h) at 1250 rpm at room temperature. After CDI activation, the samples were filtered on a membrane filter and then washed with CH_2_Cl_2_. To the CDI-activated SWCNT_COOH_, a propane-1,3-diamine solution (10 μL in 3 mL of distilled water) was added, followed by overnight incubation at 1250 rpm at room temperature. The samples were filtered on a membrane filter and washed with distilled water. In the final step, the propane-1,3-diamine functionalized supports were immersed in a solution of 25 mM *N*-maleoyl-β-alanine, 60 mM *N*-hydroxysuccinimide (NHS), and 100 mM 1-ethyl-3-(3-dimethylaminopropyl)carbodiimide (EDAC) in 3 mL dimethylformamide (DMF) and incubated for 16 h at 1250 rpm at room temperature, followed by membrane filtration and washing with acetonitrile and water. The support functionalized with maleimide groups was removed from the membrane filter and resuspended in 3 mL solution containing 1 mg of the corresponding *Pc*PAL variants (S390C, S542C, S614C and S707C) in 100 mM Tris·HCl at pH 7.5. The resulting mixtures were shaken at room temperature at 1250 rpm for 4 h, followed by filtration, washing with distilled water (3 × 3 mL) and freeze-drying, resulting in the corresponding immobilized preparations (SWCNT_COOH_-SS-PALs). The amount of the enzyme immobilized on the support was determined by protein concentration measurements of the protein solutions before and after the final thiol–maleimide coupling steps, using the Bradford method.

### Site-specific immobilization onto SWCNT_NH_2__

20 mg of SWCNT_NH_2__ was incubated with 3 mL of maleimide solution (25 mM *N*-maleoyl-β-alanine, 60 mM NHS and 100 mM EDAC in 3 mL DMF) for 16 h at 1250 rpm at room temperature, followed by membrane filtering and washing with acetonitrile and water. The obtained maleimide functionalized SWCNT_NH_2__ was removed from the membrane filter and resuspended in 3 mL solution containing 1 mg of the corresponding *Pc*PAL variants (S390C, S542C, S614C and S707C) in 100 mM Tris·HCl at pH 7.5. The resulting mixtures were incubated at room temperature at 1250 rpm for 4 h, followed by filtration, washing with distilled water (3 × 3 mL) and freeze-drying, resulting in the corresponding immobilized preparations (SWCNT_NH_2__-SS-PALs). The amount of the enzyme immobilized on the support was determined by protein concentration measurements of the protein solutions before and after the final thiol–maleimide coupling steps, using the Bradford method.

### Site-specific immobilization onto SWCNT_NH_2__ – different enzyme loadings

The preparation and functionalization of the support were performed as described above, while 10 mg of the functionalized support was resuspended in 3 mL solution containing different amounts (0.6 mg, 0.9 mg, 1.3 mg, 1.8 mg, 2.3 mg, 3.1 mg, 4.7 mg) of the *Pc*PAL S614C variant in 100 mM Tris·HCl at pH 7.5. The amount of the enzyme immobilized on the support was determined by similar protein concentration measurements.

Site-specific immobilization of the double mutant *Pc*PALs was performed as described above using a 3 mL enzyme solution containing 1.3 mg of the *Pc*PAL variant.

### Covalent, non-specific immobilization procedure using the SWCNT_COOH_ or SWCNT_NH_2__ supports

The covalent binding of 1 mg of *wtPc*PAL to 20 mg of SWCNT_COOH_ or SWCNT_NH_2__*via* a glycerol diglycidyl ether-based linker was performed using the same conditions as described in priorly developed methods.^18,20^ The amount of the non-specifically immobilized *Pc*PAL was determined from protein concentration measurements of the protein solutions before and after the immobilization, using the Bradford method.

Covalent, non-specific immobilization of the double mutant PALs was performed as described above, excepting the use of 1.3 mg of *Pc*PAL L134A and *Pc*PAL I460V and 10 mg of SWCNT_NH_2__.

### Ammonia elimination reactions

The ammonia elimination reactions were performed in 2 mL glass bottles (vials), containing 1 mg of the correspondingly immobilized *Pc*PAL-biocatalysts (SWCNT_NH_2__-SS-PALs and SWCNT_COOH_-SS-PALs) suspended in 1 mL Tris-buffer (20 mM Tris·HCl, 100 mM NaCl, pH 8.8) with a 4 mM d,l-Phe concentration. The reaction mixtures were incubated at room temperature at 750 rpm in a Heidolph Vibramax 110 incubator for different reaction times. For determination of conversion values, samples of 50 μL were removed from the reaction mixture, quenched by adding an equal volume of MeOH, vortexed and centrifuged (13 300 rpm, 17 000*g*, 1 min). The supernatant was filtered through a 0.2 μM modified nylon membrane filter and analyzed by high performance liquid chromatography (HPLC).

### Ammonia addition reactions – general procedure

The ammonia addition reactions were performed using 2 mL glass bottles (vials), containing the immobilized *Pc*PAL-biocatalysts, SWCNT_NH_2__-SS-PALs or SWCNT_COOH_-SS-PALs, prepared as described above. For the biotransformations, 1 mg immobilized biocatalyst was suspended in 1 mL of ammonia containing buffer, 2, 4, and 6 M NH_4_OH at pH 10 (adjusted with CO_2_) or 1, 2, 3, and 4 M NH_2_CO_2_NH_4_ (pH 9.6–10 without adjustment), with a 2 mM *trans*-cinnamic acid concentration. The reaction mixtures were incubated at room temperature at 750 rpm in a Heidolph Vibramax 110 platform shaker for the specified reaction times. For determination of conversion values, samples of 50 μL were removed from the reaction mixture, quenched by adding an equal volume of MeOH, vortexed and centrifuged (13 300 rpm, 17 000*g*, 1 min). The supernatant was filtered through a 0.2 μm modified nylon membrane filter and analyzed by high performance liquid chromatography (HPLC).

### Ammonia addition reactions – effect of the enzyme load

The addition reactions were performed under the conditions of the general procedure, excepting the use of 1 mg biocatalyst with different enzyme loadings (5.6 × 10^−2^, 8.8 × 10^−2^, 12.8 × 10^−2^, 17.3 × 10^−2^, 22.1 × 10^−2^, 30.9 × 10^−2^ and 46.6 × 10^−2^ mg protein per mg support) and 6 M NH_4_OH at pH 10 (adjusted with CO_2_) as the ammonia source. The reaction mixtures were incubated at room temperature, at 1250 rpm in a Heidolph Multi Reax shaker, for specified reaction times. For conversion determinations, samples from the reactions were similarly processed to those in the general procedure.

### Ammonia addition reactions – effect of the ammonia source

The reactions were performed under the conditions of the general procedure, excepting the use of 1 mg biocatalyst with the optimal enzyme loading and 2, 4, and 6 M NH_4_OH at pH 10 (adjusted with CO_2_) and 1, 2, 3, and 4 M NH_2_CO_2_NH_4_ at pH 9.6 as the ammonia source. The reaction mixtures were incubated at room temperature, at 1250 rpm in a Heidolph Multi Reax shaker, for a 16 h reaction time. For conversion determinations, samples from the reactions were similarly processed to those in the general procedure.

### Ammonia addition reactions – biocatalyst recycling procedure

The reactions were performed under the conditions of the general procedure, excepting the use of 1 mg biocatalyst with the optimal enzyme loading in 1.5 mL polypropylene spin columns as reaction vials and 6 M NH_4_OH at pH 10 (adjusted with CO_2_) and 2, 3, or 4 M NH_2_CO_2_NH_4_ at pH 9.6 as the ammonia source. The reaction mixtures were incubated at room temperature, at 1250 rpm in a Heidolph Multi Reax shaker, for specified reaction times. The spin columns were centrifuged after each cycle and the biocatalysts were washed with the corresponding ammonia and ammonium carbamate solutions. For conversion determinations, samples from the reactions were similarly processed to those in the general procedure.

### Ammonia addition reactions – temperature screening

The reactions were performed under the conditions of the general procedure, excepting the use of 1 mg biocatalyst with the optimal enzyme loading in 1.5 mL polypropylene spin columns as reaction vials and 6 M NH_4_OH at pH 10 (adjusted with CO_2_) and 2, 3, and 4 M NH_2_CO_2_NH_4_ at pH 9.6 as the ammonia source. The reaction mixtures were incubated at different temperatures (20–60 °C) at 1200 rpm, in an Eppendorf ThermoMixer C, for specified reaction times. For conversion determinations, samples from the reactions were similarly processed to those in the general procedure.

### Ammonia addition reactions – optimal conditions

The reactions were performed using 2 mL glass bottles (vials), containing 1 mg of the immobilized *Pc*PAL-biocatalysts with the optimal enzyme loading and 3 M NH_2_CO_2_NH_4_ at pH 9.6 as the ammonia source. The reaction mixtures were incubated at 40 °C at 750 rpm in a Heidolph Vibramax 110 platform shaker for the specified reaction times.

### Ammonia addition reactions for the production of l-phenylalanine analogues

The ammonia addition reactions were performed in 2 mL glass bottles (vials), containing 0.7 mg of the corresponding immobilized biocatalyst with the optimal enzyme load in 700 μL of 2 mM substrate (3-methoxycinnamic acid, 2-(trifluoromethyl)cinnamic acid, 2-methoxycinnamic acid and 4-bromocinnamic acid) solution in 3 M NH_2_CO_2_NH_4_ buffer (pH 9.6–10 without adjustment). The reaction mixtures were incubated at 40 °C at 750 rpm in a Heidolph Vibramax 110 platform. The determination of conversion values was performed by similar HPLC analyses.

## Conclusions

This study focused on the site-specific immobilization of the well-studied phenylalanine ammonia-lyase originating from *Petroselinum crispum* in order to provide a biocatalyst with high efficiency and operational stability in the ammonia addition reaction of cinnamic acid derivatives, which allows the production of l-phenylalanine analogues. The priorly reported, largely extended substrate scope and high catalytic efficiency of *Pc*PAL justify its selection as a representative of the PAL family.

The immobilization procedure employed maleimide–Cys coupling, while the immobilization site within the surface of *Pc*PAL has been optimized using several mutant variants. As a support for the protein binding, amino-functionalized carbon nanotubes proved to be effective. The immobilized biocatalyst SWCNT_NH_2__-SS-*Pc*PAL was further optimized based on the enzyme load, while several reaction conditions have been also tailored in order to ensure high productivity and operational stability of the biocatalysts. Under the optimized conditions, excellent ∼90% conversions of cinnamic acid into l-Phe have been obtained in less than 10 h reaction times, which have been maintained over several >7 reaction cycles. The developed immobilization protocol was applied also for engineered *Pc*PAL variants, L134A and I460V, of enhanced catalytic activity towards non-natural substrates, with the novel biocatalysts demonstrating superior catalytic efficiency to that of their non-specifically immobilized counterparts in the ammonia addition reactions of several *trans*-cinnamic acid derivatives.

These results show that the site-specific feature of the binding minimizes unwanted interactions on the enzyme's surface and optimizes the thermodynamic stability of the protein and, in our case, provides robust immobilized PALs with unique high recyclability in valuable ammonia addition reactions, useful for the production of enantiomerically enriched l-phenylalanines of high synthetic interest.

## Author contributions

K. B. and M. E. M. contributed to this work equally. M. E. M. and L. C. B. designed the experiments. M. E. M. performed the site-directed mutagenesis, protein isolation and initial biocatalyst screenings, while K. B. performed the immobilization/biotransformation optimization steps. C. L. N. was responsible for protein structural analysis, graphical data preparations and other computational support. L. C. B. conceived the project and was responsible for funding together with M. E. M. M. I. T., C. P. and L. C. B. inspected all data and wrote the paper together with K. B. and M. E. M. All authors reviewed this manuscript.

## Conflicts of interest

There are no conflicts to declare.

## Supplementary Material

CY-011-D1CY00195G-s001
