# Supplementary material for: Robust, site-specifically immobilized phenylalanine ammonia-lyases for the enantioselective ammonia addition of cinnamic acids
Source: Catal Sci Technol. 2021 Jun 29;11(16):5553–63. doi: 10.1039/d1cy00195g (PMC8504149; doi:10.1039/d1cy00195g)
Supplement: CY-011-D1CY00195G-s001 [file CY-011-D1CY00195G-s001.pdf]

Electronic Supplementary Material (ESI) for Catalysis Science & Technology.
This journal is © The Royal Society of Chemistry 2021

Supporting information

Robust, site-specifically immobilized phenylalanine ammonia-lyases for the enantioselective ammonia addition to cinnamic acids

Authors: Krisztina Boros[†], Mădălina Elena Moisă[†], Csaba Levente Nagy, Csaba Paizs, Monica Ioana Toșa, László Csaba Bencze*

[†]these authors contributed equally to this work

*Address for correspondence: Dr. László Csaba Bencze, cslbencze@chem.ubbcluj.ro

[1]Enzymology and Applied Biocatalysis Research Center, Faculty of Chemistry and Chemical Engineering, Babeș-Bolyai, University of Cluj-Napoca, Arany János Str. 11, RO-400028 Cluj-Napoca, Romania

Table of contents

1. Site-directed mutagenesis 3
2. Activity measurements for the purified enzymes 3
   a. Specific activities 3
   b. Conversion values in the ammonia elimination and addition reactions 2
3. Thermal denaturing profiles 2
4. Site-specific immobilization onto SWCNTs – immobilization yields and enzyme loads 3
5. Determination of conversion and enantiomeric excess values by HPLC 3
6. Structural overview of residues involved in the site-specific immobilization 6
7. Biotransformation optimization 6
   7.1. Biocatalyst screening 6
   7.2. Specific activities of the immobilized enzymes and conversion values in the ammonia addition reaction 6
   7.3. Effect of increased amount of biocatalyst on the conversion values 8
   7.4. Recycling stability of the $SWCNT_{NH2}$-SS-*Pc*PAL and $SWCNT_{NH2}$-GDE-*wtPc*PAL in ammonium carbamate solution 8
   7.5. Recycling stability of the $SWCNT_{NH2}$-SS-*Pc*PAL in NH_4OH-based reaction medium 9
8. Time conversion profile 9
9. References 10

1. Site-directed mutagenesis

*Pc*PAL mutant variants S390C, S542C, S614C, S707C were obtained through site-directed mutagenesis using the *Pc*PAL C704S/C716S cloned in pET-19b expression vector as template. The PCR reaction contained 2 ng of template DNA, 2 µM solution of primer pair (**Table S1**), 200 µM dNTPs, 2 U/µL of Phusion High-Fidelity DNA polymerase and 10 µL of 5X Phusion HF Buffer, filled up to 50 µL with water. The PCR cycles were initiated at 95 °C for 3 minutes, followed by 30 amplification cycles. Each amplification cycle consisted of denaturation at 95 °C for 1 minute, annealing at temperature of $T_{m\ no}$-5 °C for 1 min and extension at 72 °C for 8 minutes. The PCR cycles were finished with a final annealing step at $T_{m\ pp}$-5 °C for 1 minute and a final extension step at 72 °C for 30 minutes. Volumes of 10 µL from each PCR reaction were used for analysis by agarose gel electrophoresis. Next, the PCR product was digested with 5 units of DpnI restriction enzyme at 37 °C for 2 h to remove the template DNA. An aliquot of 5 µL from the above digested product was transformed into 100 µL of *E. coli* XL1-Blue chemically competent cells by heat shock. The transformed cells were plated on a Luria-Bertani (LB) plate containing 50 µg/mL carbenicillin and 10 µg/mL tetracycline and incubated at 37 °C for 16 h. The resulted colonies were tested for the presence of the plasmid using colony PCR and two of the positive colonies from each plate were grown and the plasmid DNA was isolated. To verify the mutations, DNA sequencing was carried out using the sequencing service of Biomi (Gödöllő, Hungary).

In case of mutants *Pc*PAL S542C and *Pc*PAL S707C the optimization of the PCR reactions was required in order to successfully accomplish the site-directed mutagenesis. 4 ng of the template DNA and additional 3% DMSO were added to the PCR reaction, and the final concentration of the primer pair was increased to 3 µM. The temperature of the annealing step in the PCR cycles was risen to 70 °C.

In case of *Pc*PAL double mutant variants (*Pc*PAL L134A/S614C and *Pc*PAL I460V/S614C) the site-directed mutagenesis was realized under the same conditions as mentioned above, excepting the use of the previously obtained *Pc*PAL L134A and *Pc*PAL I460V variants[1] as template DNA.

Table S1. Primers designed for PCR-based site-directed mutagenesis of the Ser residues to Cys in the four selected positions. The primer design and mutagenesis were performed using an earlier described procedure[2].

Primer name	Sequence	$T_{m\ pp}$ (℃)	$T_{m\ no}$ (℃)	$T_{m\ full}$ (℃)	T_A (℃)	T_{FA} (℃)
**S390C/FP**	*5’*GACGTA**TGC**AGAAAC*AAGGCCATTCATGGAG3’*	49.4	67.7	70.1	62	45
**S390C/RP**	5’GTTTCT**GCA**TACGT*CAATCAAGGGGTTGTCG3*’	49.4	67.2	70.0		
**S542C/FP**	*5’*GTATCC**TGC**GTAGC*CAAGAGAGTATTGACTATGGG3’*	50.2	56.2	70.7	70	45
**S542C/RP**	*5’*GCTAC**GCA**GGATAC*AGTGTTCTTTACAGTGGAC3’*	50.2	55.9	70.3		
**S614C/FP**	*5’*GTCCACT**TGC**ATTTTCC*AGAAGATTGCCACTTTCGAAG3’*	54.8	59.9	72.3	55	50
**S614C/RP**	*5’*GGAAAAT**GCA**AGTGGAC*AAGTTTCTTTCGTTGTCTCCG3’*	54.8	60.0	72.3		
**S707C/FP**	*5’*CTTGGAA**TGC**TGGAAC*GGAGCCCCCTTGCC3’*	53.4	60.9	76.8	70	48
**S707C/RP**	*5’*GTTCCA**GCA**TTCCAAG*GATTCCAACAAGGGGTCAATAATTTC3’*	53.4	61.7	72.8		

2. Activity measurements for the purified enzymes

a. Specific activities

The activity assays of the purified proteins were performed in Tris buffer, pH 8.8 (100 mM Tris.HCl, 120 mM NaCl) with 2 mM substrate concentration by adding 10 µg of the corresponding purified *Pc*PAL variant in 200 µL final reaction volume, at 30 °C, by monitoring the production of *trans*-cinnamic acid at 290 nm for 5 minutes using Tecan Infinite Spark 10M UV plate reader.

Table S2. Specific activities of the purified *Pc*PAL variants.

Specific activities (μmol/mg E/min)	Relative enzyme activities (%)	Enzyme
0.296	103.88	*Pc*PAL S390C
0.279	97.86	*Pc*PAL S542C
0.263	92.03	*Pc*PAL S614C
0.332	116.20	*Pc*PAL S707C
0.285	100	*Wt-Pc*PAL

b. Conversion values in the ammonia elimination and addition reactions

In the reactions with isolated enzymes the same protein quantities were used as in the ammonia elimination and addition reactions with immobilized enzymes presented in **Fig. 4** (see Experimental part, general procedures):

The ammonia elimination reactions were performed in 1.5 mL Eppendorf tubes, containing 0.049 mg/mL final concentration of the *Pc*PAL variants in 0.5 mL Tris-buffer (20 mM Tris.HCl, 100 mM NaCl, pH 8) at 4 mM D,L-Phe concentration. The reaction mixtures were incubated at 30 °C, at 750 rpm in an Eppendorf ThermoMixer C for different reaction times. For determination of conversion values samples of 50 μL were removed from the reaction mixture, quenched by adding an equal volume of MeOH, vortexed and centrifuged (13300 rpm, 17000 × g, 1 min). The supernatant was filtered through a 0.2 μm modified nylon membrane filter and analyzed by high performance liquid chromatography (HPLC).

The ammonia addition reactions were performed using 1.5 mL Eppendorf tubes, containing the isolated, soluble *Pc*PAL variants. For the biotransformations 0.049 mg/mL final concentration of the purified enzymes was used in 6 M NH_4OH (pH 10) buffer with 2 mM *trans*-cinnamic acid concentration. The reaction mixtures were incubated at 30 °C, at 750 rpm in an Eppendorf ThermoMixer C for the specified reaction times. For conversion determinations samples from the reactions were similarly processed as noted above.

Table S3. Results supporting that isolated, soluble *Pc*PAL variants have similar conversion-based activities as the *wtPc*PAL.

Soluble enzymes	Conversion (%)					
	Addition			Elimination		
	1 h	2 h	4 h	1 h	2 h	4 h
wt-*Pc*PAL	72.9	82.2	85.9	38.6	38.5	38.8
*Pc*PAL S390C	64.5	76.0	85.9	31.3	35.6	39.0
*Pc*PAL S542C	64.7	76.2	85.6	32.8	39.2	38.4
*Pc*PAL S614C	65.4	77.8	85.6	29.9	35.6	37.9
*Pc*PAL S707C	67.7	79.6	85.5	35.5	38.3	39.4

3. Thermal denaturing profiles

The thermal unfolding profiles of the *Pc*PAL variants were determined by real-time protein unfolding experiments performed in a BioRad CFX96 Real-Time Thermal Cycler using the ROX fluorescence filter. Samples of 45 μL consisting of *Pc*PAL variants (5 μM) in 20 mM Tris, 100 mM NaCl pH 8 buffer were mixed with 5 μL of SYPRO Orange Protein Gel Stain (Thermo Fisher Scietific) 200X solution (prepared

from a 5000X solution using the previously mentioned Tris buffer solution) in a BioRad Hard-Shell 96-well microplate. A negative control (NC) was similarly prepared by mixing 45 µL of the Tris buffer solution with 5 µL of SYPRO Orange 200X dye. The unfolding of *Pc*PAL enzymes was measured during heating between 20-95 °C, with an increment of 1 °C. For determination of the protein melting points (T_m) the first derivatives of the fluorescence emissions were represented as a function of temperature (-d(RFU)/dT). The obtained thermal denaturing profiles are presented in **Fig. S1**.

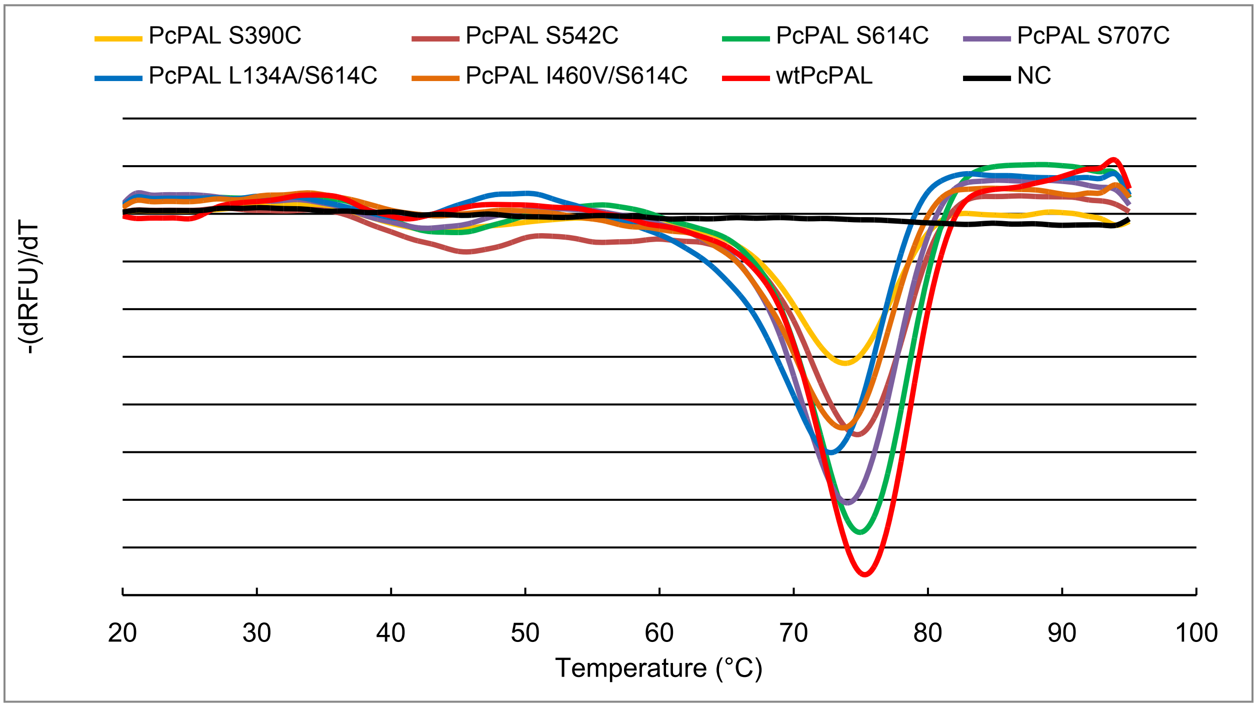

Figure S1: Thermal denaturing profile of the purified *Pc*PAL variants.

4. Site-specific immobilization onto SWCNTs – immobilization yields and enzyme loads

Table S4. Enzyme immobilization yields in case of site-specific immobilizations onto both types of SWCNT support.

Enzyme	$SWCNT_{COOH}$		$SWCNT_{NH2}$	
	bound	mg enzyme/mg support	bound	mg enzyme/mg support
**wt*Pc*PAL**	98.7%	4.9×10^{-2}	98.1%	4.9×10^{-2}
**S390C**	98.6%	4.9×10^{-2}	98.6%	4.9×10^{-2}
**S542C**	97.6%	4.9×10^{-2}	96.9%	4.9×10^{-2}
**S614C**	97.3%	4.9×10^{-2}	97.7%	4.9×10^{-2}
**S707C**	97.7%	4.9×10^{-2}	97.4%	4.9×10^{-2}

5. Determination of conversion and enantiomeric excess values by HPLC

a) Reversed-phase high-performance liquid chromatography was used in order to determine the conversions of the *Pc*PAL-catalyzed ammonia elimination and ammonia addition reactions. The analyses were conducted with Agilent (Santa Clara, CA, USA) 1200 and 1100 systems. The samples, retrieved from the biotransformations and prepared accordingly to the descriptions from the experimental part,

were injected onto a Gemini NX-C18 column (150×4.5 mm; 5 μm) and eluted with a flow rate of 1.0 mL/min at 25 °C using a gradient of the mobile phase consisting of NH_4OH buffer (0.1 M, pH 9.0) and MeOH. The conversions were determined using the relative response factor of *trans*-cinnamic acids compared to L-phenylalanine derivatives (**Table S5**), that was determined through HPLC analysis of several mixtures of different and known molar ratios of the two reaction partners.

Table S5. HPLC methods and response factors used for the conversion value determination.

Substrate	Mobile phase* gradient (%B)	Retention time (min)		Response factor	Temperature (°C)
		L-amino acid product	Substrate	substrate vs. L-amino acid	
trans-cinnamic acid	10 to 40 in 8 min	4.3	7.9	14.615	25
3-methoxycinnamic acid	25 to 32 in 8 min	3.5	6.3	2.869	25
2-methoxycinnamic acid		3.8	6.6	4.156	
2-(trifluoromethyl) cinnamic acid	35 to 50 in 6 min	4.6	6.0	2.119	30
4-bromocinnamic acid	28 to 50 in 8 min	5.5	8.5	1.230	30

*Mobile phase: a. NH_4OH buffer (0.1 M, pH 9.0), b. MeOH; flow rate: 1.0 mL/min, using Gemini NX-C18 column (150×4.5 mm; 5 μm).The wavelength used for UV detection was 220 nm.

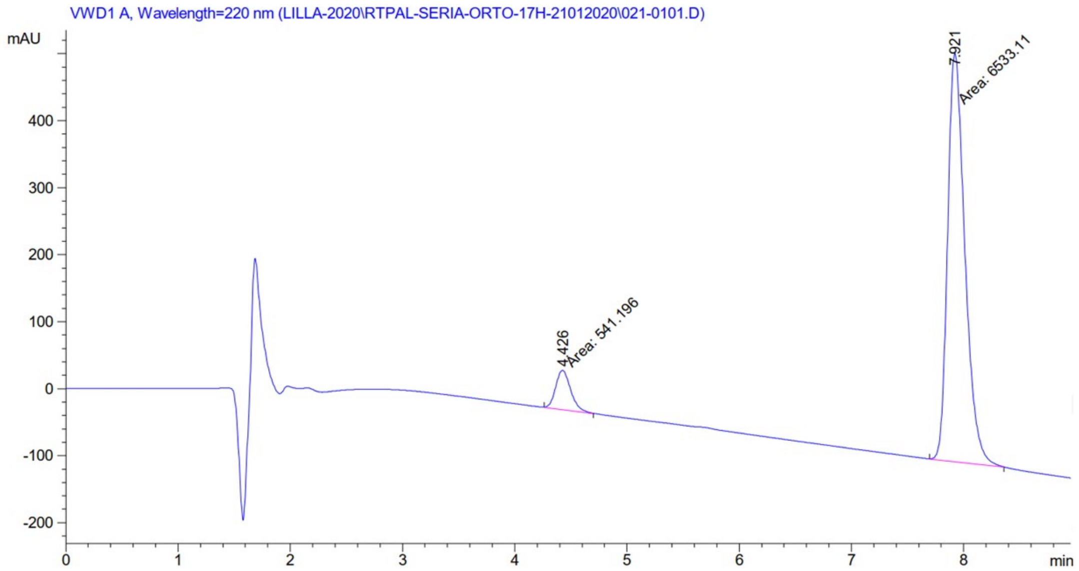

Figure S2: HPLC chromatogram from the separation of D,L-phenylalanine and *trans*-cinnamic acid at 1:1 molar *ratio*.

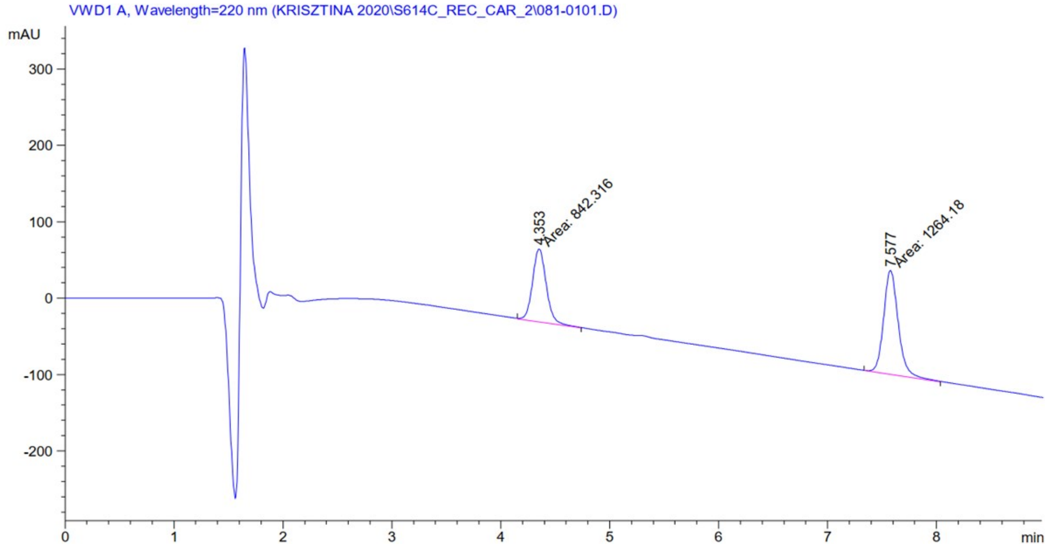

Figure S3: HPLC chromatogram of samples taken after 12 hours reaction time from the ammonia addition onto *trans*-cinnamic acid performed under the optimal conditions (40 °C, vibrational stirring at 750 rpm of the 1 mL 3 M $NH_2CO_2NH_4$ reaction solution containing 2 mM cinnamic acid and 1 mg of biocatalyst with a load of 0.12 mg enzyme/mg support), corresponding to conversion value of 90%.

b) The enantiomeric excess values of L-Phe obtained from the ammonia addition reactions were determined employing chiral HPLC. Firstly, the separation of *rac*-phenylalanine was developed on Crownpak CR-I (+) chiral column (150×3 mm; 5 μm), using as mobile phase a mixture of $HClO_4$ (pH=1.5) and ACN at 80:20 volume *ratio*, at 0.4 mL/min flow rate and 25 °C (**Figure S4**). The absolute configuration of the eluted enantiomers was assessed from their elution order from the chiral CROWNPAK CR-I (+) column according to the manufacturer's instructions and was also confirmed by the obtained retention times for commercial D- and L-Phe standards ($Rt_{D\text{-}Phe}$ = 3 min, $Rt_{L\text{-}Phe}$ = 5 min).

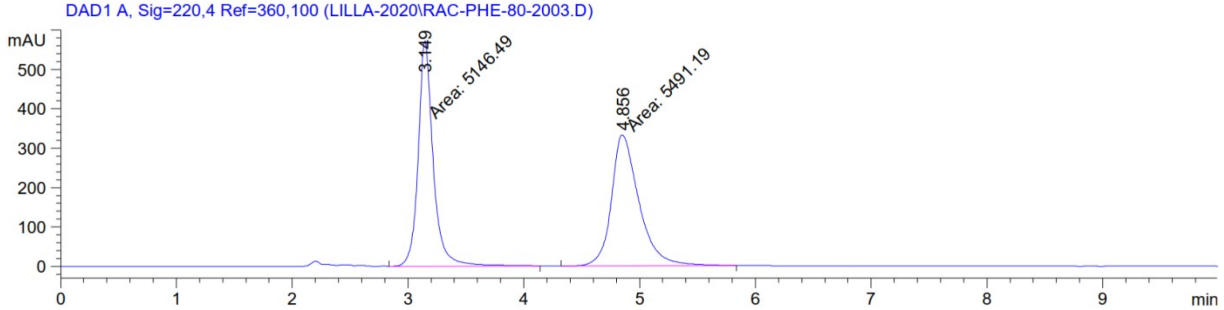

Figure S4: HPLC chromatogram of D,L-phenylalanine on Crownpak CR-I (+) chiral column.

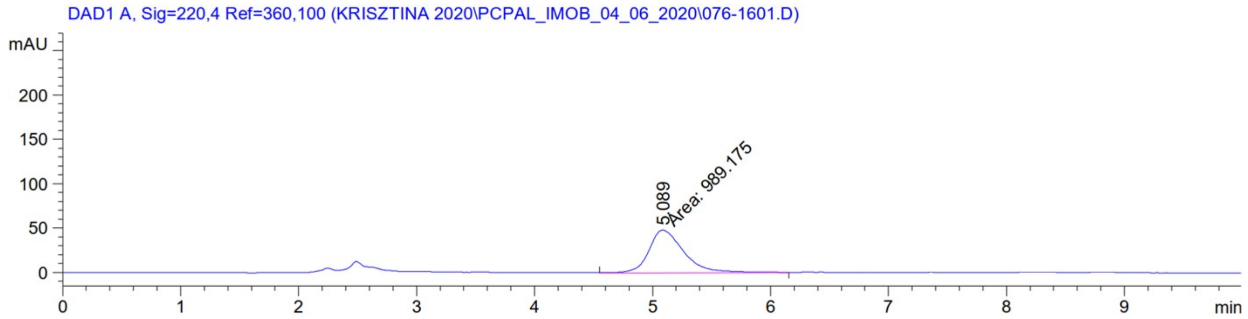

Figure S5: HPLC chromatogram of the $SWCNT_{NH2}$-SS-PAL/S614C catalyzed ammonia addition reaction onto *trans*-cinnamic acid performed under the conditions of the first batch (room temperature, vibrational stirring at 750 rpm of the 1 mL 6 M NH_4OH reaction solution containing 2 mM cinnamic acid and 1 mg of biocatalyst with a load of 0.05 mg enzyme/mg support) after 14 h reaction time, showing an ee value >99% for the produced L-phenylalanine.

6. Structural overview of residues involved in the site-specific immobilization

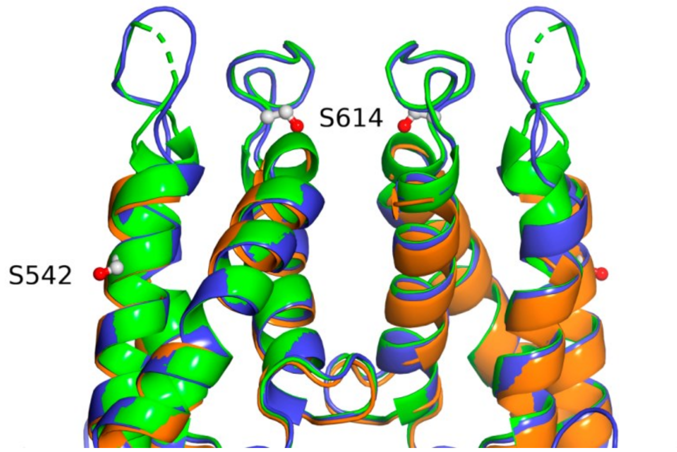

Figure S6: Overlay of *Pc*PAL structures 6F6T (green), 1W27 (blue) and 6RGS (orange) focusing on the loop containing residue 614, connecting two helices at the top of the additional insertion domain of *Pc*PAL. In the crystal structure of the *Pc*PAL I460V variant (6RGS, orange), *p*-MeO-cinnamic acid, a substrate analogue for the ammonia addition reaction is the ligand, and the loop containing S614 showed insufficient electron density. In *Pc*PAL structures complexed with inhibitor-type ligands such as DTT (1W27, blue) and S-APPA (6F6T, green) the structure of the loop containing resiude 614 is defined.

7. Biotransformation optimization

7.1. Biocatalyst screening

Table S6. Results supporting that *Pc*PALs immobilized on $SWCNT_{NH2}$ show higher activity in the ammonia addition reaction than the same enzymes immobilized on $SWCNT_{COOH}$.

***Pc*PAL/S390C and wt-*Pc*PAL on different SWCNTs**	**Conversion $_{24\,h}$ (%)**	
	**Ammonia elimination***	**Ammonia addition***
**$SWCNT_{COOH}$ -SS-*Pc*PAL/S390C**	**22.2**	14.2
**$SWCNT_{NH2}$-SS-*Pc*PAL/S390C**	9.8	**25.5**

**$SWCNT_{COOH}$ -GDE-wt*Pc*PAL**	**11.7**	8.2
**$SWCNT_{NH2}$-GDE-wt*Pc*PAL**	3.6	**18.9**

*The ammonia elimination reactions were performed at room temperature, at 750 rpm (vibrational stirring), in 1 mL Tris-buffer (20 mM Tris.HCl, 100 mM NaCl, pH 8.8,) at 4 mM D,L-Phe concentration using 1 mg of biocatalyst (*Pc*PAL site-specifically immobilized through the Cys390 residue and the non-specifically, covalently immobilized wt-*Pc*PAL). The ammonia addition reactions were performed at room temperature, at 750 rpm (vibrational stirring), in 1 mL 6 M NH_4OH reaction solution containing 2 mM substrate, using 1 mg of biocatalysts (*Pc*PAL site-specifically immobilized through the Cys390 residue and the non-specifically, covalently immobilized wt-*Pc*PAL).

7.2. Specific activities of the immobilized enzymes and conversion values in the ammonia addition reaction

Despite our efforts, kinetic measurements for the ammonia addition reactions were not successful, probably due to the high background caused by the elevated ammonia concentration and/or the high UV absorbance of the *trans*-cinnamic acid substrate. Notable, that in our previous studies[1,3] we met similar obstacles for the kinetic measurements of the ammonia additions reactions with purified, soluble enzymes, hindering the calculation of specific activities within this important reaction route.

Accordingly the determination of specific activities of the $SWCNT_{NH2}$-SS-PALs were limited for the ammonia elimination reaction, where based on their conversion-activity they underperform in comparison with the ammonia additions (see **Table S6**). Furthermore, we can rely also on the conversion-based activities: as one can see in **Table S3** soluble, purified enzymes provided similar conversions after 4 h reaction time, while the immobilized variants (**Table S7**) clearly possess different activities within the ammonia addition reactions.

Table S7. Specific activities of the immobilized biocatalysts within the ammonia elimination from L-Phe and HPLC conversion values in the ammonia addition reaction onto *trans*-cinnamic acid at different reaction times.

**Biocatalyst**	**Specific activities in the ammonia elimination (µmol/mg E/min)***	**Conversions in the ammonia addition reaction (%)**			**Productivity in the ammonia addition reaction (µmol/g/min)****
		**2 h**	**4 h**	**20 h**	
$SwCNT_{NH2}$-SS-*Pc*PAL	0.041	40.1	66.1	89.9	6.04
$SwCNT_{NH2}$-GDE-*wtPc*PAL	0.015	13.1	28.8	87.7	2.40

*mg E refers to the fact that specific activities were calculated considering the enzyme quantity, not the quantity of the immobilized biocatalyst (enzyme and support).

** calculated using the equation $r = \frac{n_p}{t * m_b}$, similarly as reported by Bartha-Vári *et al.*[4], where m_b refers to the mass of the immobilized biocatalyst (enzyme and support).

Experimental procedure for the determination of specific activities and conversion-activities of the immobilized enzymes:

The ammonia elimination reactions of L-Phe were performed in 2 mL glass bottles (vials), containing 1 mg of the immobilized *Pc*PAL-biocatalysts ($SWCNT_{NH2}$-SS-PAL, $SWCNT_{NH2}$-GDE-PAL) with optimal 0.13 mg enzyme load in 1 mL Tris-buffer (20 mM Tris.HCl, 100 mM NaCl, pH 8.8) at 2 mM L-Phe concentration. The reaction mixtures were incubated at 30 °C, at 750 rpm in a Heidolph Vibramax 110 incubator. In every 2 minutes an 80 µL sample was removed from the reaction mixture, diluted to 200 µL final volume with Tris-buffer (20 mM Tris.HCl, 100 mM NaCl, pH 8) and the production of *trans*-cinnamic acid was monitored at 290 nm.

The conversion based activities were similarly determined as described within the main manuscript, (*Experimental part, section: ammonia additions under optimal conditions*) excepting the use of 1.5 mL

Eppendorf tubes as reaction vials. The samples were incubated at 900 rpm in an Eppendorf ThermoMixer C for the specified reaction times. For conversion determinations samples from the reactions were similarly processed as noted above, in section 2.

7.3. Effect of increased amount of biocatalyst on the conversion values

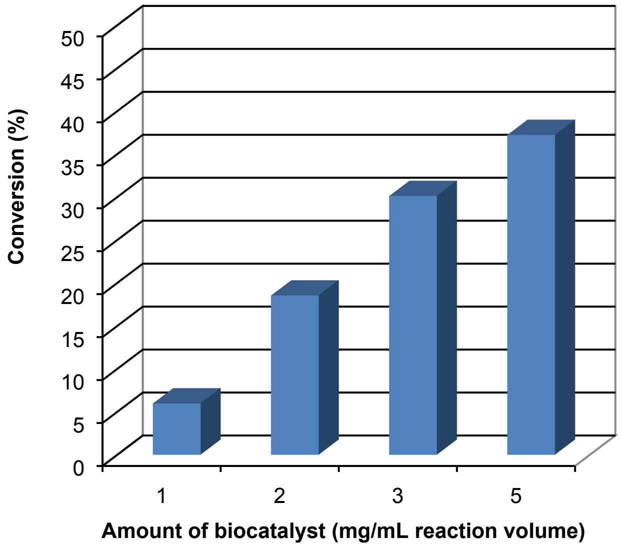

Figure S7: Conversions of the ammonia addition reactions performed at room temperature, at 750 rpm (vibrational stirring), using different amounts of biocatalyst (SWCNT$_{NH2}$-SS-*Pc*PAL/S614C with a load of 0.05 mg enzyme/mg support) in 1 mL 6 M NH_4OH solution containing 2 mM substrate after 18,5 h reaction time.

7.4. Recycling stability of the SWCNT$_{NH2}$-SS-*Pc*PAL and SWCNT$_{NH2}$-GDE-*wtPc*PAL in ammonium carbamate solution

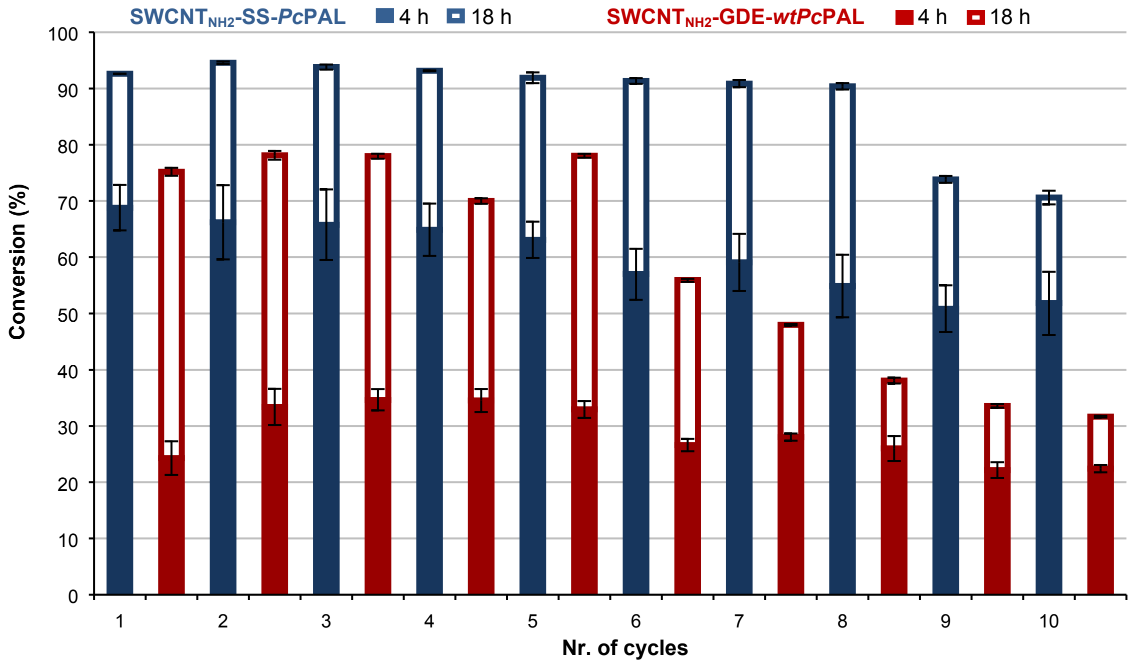

Fig. S8: Recyclability of $SWCNT_{NH2}$-SS-*Pc*PAL and $SWCNT_{NH2}$-GDE-*wtPc*PAL in the ammonia addition reaction of cinnamic acid using the optimal reaction conditions, including ammonium carbamate (3 M; pH 9.6–10.0) as ammonia source and the optimal enzyme load.

7.5. Recycling stability of the $SWCNT_{NH2}$-SS-*Pc*PAL in NH_4OH-based reaction medium

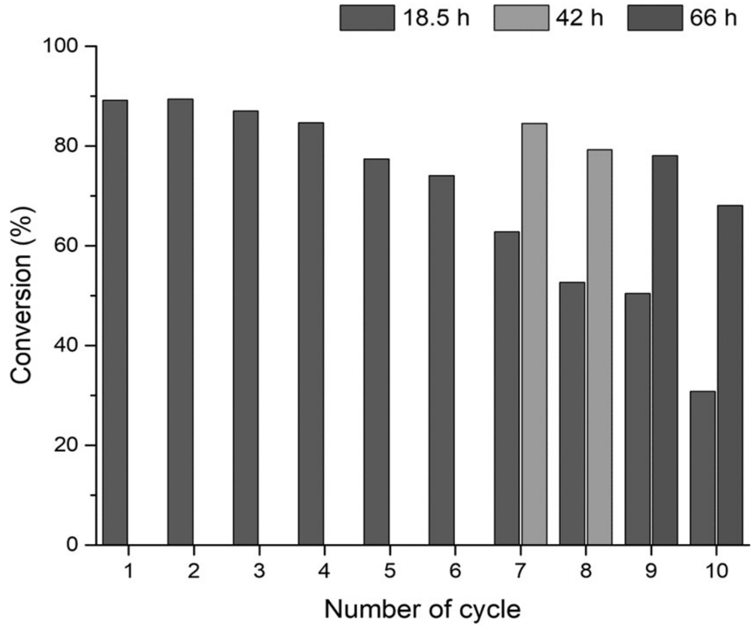

Figure S9: Recyclability of $SWCNT_{NH2}$-SS-*Pc*PAL in the ammonia addition reaction of cinnamic acid using 6 M NH_4OH (pH=10.0) as ammonia source. The reactions were performed at room temperature, at 1250 rpm, using 1 mg of biocatalyst with the optimal enzyme loading. The samples were taken at specified reaction times.

8. Time conversion profile

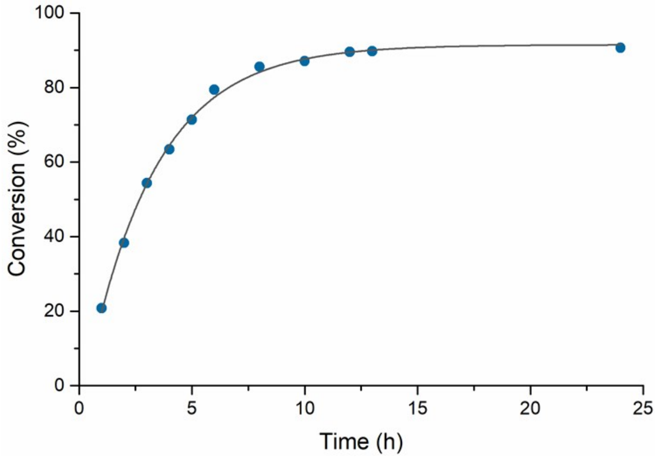

Fig. S10: Time-conversion profile of the ammonia addition reaction performed under the optimal reaction conditions: 40 °C, vibrational stirring at 750 rpm of the 3 M $NH_2CO_2NH_4$ reaction solution containing 2 mM cinnamic acid with biocatalyst ($SWCNT_{NH2}$-SS-PAL: 0.12 mg enzyme/mg support) load of 1 mg/mL reaction volume.

Experimental procedure:

1 mg biocatalyst with the optimal enzyme loading was suspended in 1 mL of 3 M $NH_2CO_2NH_4$, pH 9.6 buffer. The reaction mixture was incubated at 40 °C, at 750 rpm in a Heidolph Vibramax 110 platform shaker. For conversion determinations samples from the reaction were taken at

specified reaction times and were processed accordingly to the descriptions from the experimental part.

9. References

[1] A. Filip, E. Z. A. Nagy, S. D. Tork, G. Bánóczi, I. M. Toșa, F. D. Irimie, L. Poppe, C. Paizs, L. C. Bencze, *ChemCatChem.*, 2018, **10**, 2627

[2] H. Liu, J. H. Naismith, *BMC Biotechnol.*, 2008, **8**, 91

[3] E. Z. A. Nagy, S. D. Tork, P. A. Lang, A. Filip, F. D. Irimie, L. Poppe, I. M. Toșa, C. J. Schofield, J. Brem, C. Paizs, *ACS Catal.*, 2019, **9**, 8825

[4] J. H. Bartha-Vári, L. C. Bencze, E. Bell, L. Poppe, G. Katona, F. D. Irimie, C. Paizs, M. I. Toşa, *Period. Polytech.* Chem., 2017, **61**, 59
